# Reintubation in critically ill patients: procedural complications and implications for care

**DOI:** 10.1186/s13054-014-0730-7

**Published:** 2015-01-16

**Authors:** Jonathan Elmer, Sean Lee, Jon C Rittenberger, James Dargin, Daniel Winger, Lillian Emlet

**Affiliations:** Department of Critical Care Medicine, University of Pittsburgh School of Medicine, 3550 Terrace Street, Pittsburgh, PA 15261 USA; Department of Emergency Medicine, University of Pittsburgh School of Medicine, 3600 Forbes Avenue, Iroquois Building Suite 400A, Pittsburgh, PA 15213 USA; Department of Pulmonary & Critical Care Medicine, Lahey Medical Center & Hospital, 41 Mall Road, Burlington, 01805 MA USA; Clinical and Translational Science Institute, University of Pittsburgh, Forbes Tower Suite 7057, Pittsburgh, PA 15206 USA

## Abstract

**Introduction:**

In critically ill patients, re-intubation is common and may be a high-risk procedure. Anticipating a difficult airway and identifying high-risk patients can allow time for life-saving preparation. Unfortunately, prospective studies have not compared the difficulty or complication rates associated with reintubation in this population.

**Methods:**

We performed a secondary analysis of a prospective registry of in-hospital emergency airway management, focusing on patients that underwent multiple out-of-operating room intubations during a single hospitalization. Our main outcomes of interest were technical difficulty of intubation (number of attempts, need for adjuncts to direct laryngoscopy, best Cormack-Lehane grade and training level of final intubator) and the frequency of procedural complications (aspiration, arrhythmia, airway trauma, new hypotension, new hypoxia, esophageal intubation and cardiac arrest). We compared the cohort of reintubated patients to a matched cohort of singly intubated patients and compared each repeatedly intubated patient’s first and last intubation.

**Results:**

Our registry included 1053 patients, of which 151 patients (14%) were repeatedly intubated (median two per patient). Complications were significantly more common during last intubation compared to first (13% versus 5%, *P* = 0.02). The most common complications were hypotension (41%) and hypoxia (35%). These occurred despite no difference in any measure of technical difficultly across intubations.

**Conclusion:**

In this cohort of reintubated patients, clinically important procedural complications were significantly more common on last intubation compared to first.

## Introduction

Between 10 and 20% of critically ill patients who are extubated will be reintubated within 72 hours [1-4]. Observational data suggest that 40 to 90% of these patients show signs of laryngeal damage or edema on laryngoscopy [5-8]. Furthermore, reintubation is often performed emergently, a situation in which complication rates are as high as 5 to 24% [9-16]. Providers may therefore assume that reintubation will be more difficult or associated with more complications than the initial intubation procedure, and may approach these procedures with caution. While these assumptions might have face validity, they may not be evidence based.

We are aware of no prospective studies that directly compare the technical difficulty or procedural complication rates of reintubation with first intubation in repeatedly intubated patients. Being able to accurately anticipate the potential for a difficult intubation and identify high-risk patients can help inform the appropriate selection of medications, equipment and personnel, and allow time for patient optimization [17], which is important for clinical management and reduction of risk in these potentially high-risk intubations [18]. We present an analysis of a large, prospective registry of in-hospital airway management testing the null hypothesis that reintubations are neither more difficult nor associated with more procedural complications than first intubations in repeatedly intubated critically ill patients.

## Methods

### Patients and setting

We performed a secondary analysis of a prospective registry of in-hospital emergent airway management at the University of Pittsburgh Medical Center Presbyterian Hospital. The University of Pittsburgh Medical Center Total Quality Council and the University of Pittsburgh Internal Review Board approved all aspects of this work as institutional review board exempt, and therefore the study was conducted with waiver of informed consent. University of Pittsburgh Medical Center Presbyterian Hospital is a 792-bed tertiary care Level 1 trauma center with approximately 50,000 emergency department (ED) visits annually and 150 subspecialized ICU beds. The details of this registry have been described previously [19]. The initial registry included 1,053 patients intubated over a 10-month period in the ED, in the ICU or on hospital floors, and excluded elective intubations performed in the operating room or procedural areas. In the present study, we focused on the subgroup of patients that were intubated on multiple occasions during a single hospitalization. At our hospital, in-hospital out-of-operating room intubations are generally performed by critical care fellows, who receive extensive education in both routine and difficult airway management including didactics, case-based, simulation and cadaver-based training. Intubations in the ED are generally performed by an emergency medicine resident, who receives similar didactic, case-based and simulation training. An attending intensivist or emergency physician directly supervises all intubations and post-intubation management.

During the study period, standard medications for induction (etomidate, propofol, fentanyl and midazolam), paralysis (succinylcholine and rocuronium), topical anesthetics and vasoconstrictors were available to providers regardless of the in-patient setting. Available airway equipment included standard direct largyngoscopy (DL) blades, oral and nasal airways, as well as adjuncts to DL: a gum elastic bougie, laryngeal mask airways, an Airtraq® optical laryngoscope (Prodol Ltd., Vizcaya, Spain), fiberoptic bronchoscopy, transtracheal jet ventilation equipment, video laryngoscopes – Glidescope® (Verathon Inc., Bothell, WA, USA) in the ICU and C-MAC® (Karl Storz, Inc, El Segundo, CA, USA) in the ED – and a cricothyroidotomy kit. If needed, a difficult airway team comprised of an attending anesthesiologist, a trauma surgeon and an intensivist could respond for additional support in our mature rapid response team system.

### Data collection

Our registry includes details of consecutive in-hospital intubations performed during a 10-month quality improvement initiative that ended in July 2010. Immediately after patient management, the intubator was asked to complete a web-based data entry form built with prepopulated drop-down menus (for data elements with mutually exclusive responses), checkbox responses (for nonmutually exclusive elements) and space for additional free-text information. We recorded patient details including the intubation location, indication, operator experience and preintubation airway assessment of established predictors of difficult intubation [20]. We also recorded procedural details including the devices and adjunctive techniques used for each intubation attempt, the Cormack–Lehane grade (for DL) and any immediate procedural complications. Each data entry form was crosschecked against the electronic medical record to verify accuracy. Our previous work showed that we captured data for 98% (95% confidence interval = 97 to 99%) of eligible intubations performed during the study period [19].

### Outcomes

Our main outcomes of interest were the technical difficulty of intubation and immediate procedural complications. We operationalized technical difficulty of intubation by analyzing the number of intubation attempts prior to success (≤2 vs. ≥3), the rank of the final intubator (attending physician versus resident or fellow), use of adjuncts to DL for airway management and the best Cormack–Lehane grade reported (grade I to II vs. grade III to IV). During the study period, it was our institutional practice that trainees performed the initial intubation attempts and DL was the initial method of choice unless providers had a high index of suspicion that it might fail. We defined an intubation attempt as any manipulation of the airway with the goal of placing a definitive airway (for example, placement of a laryngoscope blade into the mouth). We classified immediate procedural complications as aspiration of gastric contents, arrhythmia, dental or upper airway trauma, new sustained hypotension (sustained postinduction systolic blood pressure <95 mmHg in a previously normotensive patient), new sustained hypoxia (sustained oxygen saturation <90% in a previously nonhypoxemic patient), esophageal intubation and cardiac arrest. We considered major complications to be cardiac arrest, hypotension and hypoxia [21].

### Statistical analysis

We performed two main analyses. First, among reintubated patients we compared the technical difficulty and rate of procedural complication for each patient’s first and last intubations. To avoid multiple hypothesis testing, our *a priori* analysis plan was to analyze and report immediate procedural complications aggregated as a binary outcome (that is, any complication vs. no complications). However, since specific complications may be of clinical interest, we also decided to report summary statistics for each complication individually. In the subgroup of patients for whom data were available, we repeated these test procedures adjusting for the total antecedent duration of mechanical ventilation and the time between extubation and subsequent reintubation, which we dichotomized as <72 hours and ≥72 hours to parallel previous studies [1-4].

Next, we tested for baseline or procedural differences between repeatedly versus singly intubated patients. To do this, we used optimal Mahalanobis calipers to match each exposed patient (that is, a reintubated patient) to two unexposed (singly intubated) patients for age and gender. We compared the baseline characteristics, difficulty of intubation and procedural complications between the first intubations in exposed (reintubated) and unexposed (singly intubated) cohorts. We considered missing data not to be at random and therefore not ignorable since we suspected that the frequency of missing data might vary depending on the individual completing the data collection form and the acuity of the intubation event. We therefore included ‘missing’ as a level for the ordinal variables in our analysis.

We used descriptive statistics to summarize baseline population characteristics and report means with standard deviations. We used generalized estimating equation models with a logit link and independent correlation structure to model dichotomous outcomes and to apply clustered robust standard errors due to the two-to-one matching in the data. We used multinomial logistic regression with a cluster term to analyze categorical outcomes with more than two categories. Multinomial logistic regression produces relative risk ratios, which are similar in concept to odds ratios. We performed our analysis using STATA Version 12 (StataCorp, College Station, TX, USA), and our matching process made use of the optmatch package in R Version 3.0.0 (R Core Team, Vienna, Austria) [22].

## Results

Our registry included 1,053 patients, of whom 151 patients (14%) underwent repeated intubations. The mean age of the reintubated patients was 61 years and 40% were female. The majority of these patients were intubated twice (59%, range 2 to 5). Most intubations (82%) were performed with DL and in the ICU (84%). A minority of intubations (8%) occurred after unplanned extubation. There was no difference in any of our prespecified measures of difficulty between first and last intubations (Table 1). However, last intubations were associated with significantly more complications than the first intubation (13% vs. 6%, *P* = 0.03). The most common complications observed on last intubation were new sustained hypotension and hypoxia (41% and 35%, respectively). We had data available to control for the duration of previous mechanical ventilation and the time from antecedent extubation to reintubation in 118 of 151 reintubated patients. In this subgroup, a longer time from antecedent extubation to reintubation was associated with an increased risk of procedural complications (odds ratio = 1.05 per week, 95% confidence interval = 1.01 to 1.10, *P* = 0.018).

**Table 1:** **Comparison of intubation characteristics and procedural complication rates between first and last intubations in repeatedly intubated patients**

**Characteristic**	**First intubation**	**Last intubation**	**Odds ratio**	***P*** **value**
	**(** ***n*** **= 151)**	**(** ***n*** **= 151)**	**(95% confidence interval)**	
Intubation location				0.18
ICU	115 (77)	127 (86)	–	
Emergency department	15 (10)	8 (5)	–	
Procedural suite	2 (1)	2 (1)	–	
Hospital floor	15 (10)	11 (7)	–	
Other	3 (2)	0 (0)	–	
Intubation indication				0.27
Respiratory distress	53 (35)	57 (39)	–	
Airway protection	35 (23)	27 (18)	–	
Hypoxia	23 (15)	38 (26)	–	
Cardiac arrest	6 (4)	4 (3)	–	
Elective (preprocedure)	11 (7)	8 (5)	–	
Shock	6 (4)	2 (1)	–	
Other	16 (11)	12 (8)	–	
Intubation attempts				
1 or 2	134 (89)	138 (91)	Reference	Reference
≥ 3	17 (11)	13 (9)	0.74 (0.36 to 1.52)	0.41
Rank of final intubator				
Fellow	98 (69)	106 (72)	Reference	Reference
Resident or other	28 (10)	29 (20)	0.96 (0.58 to 1.58)	0.86
Attending	16 (11)	13 (9)	0.75 (0.39 to 1.46)	0.40
Adjunct to DL used	16 (11)	18 (12)	1.13 (0.60 to 2.15)	0.70
Cormack–Lehane grade				
1	91 (61)	88 (58)	Reference	Reference
2	33 (22)	38 (25)	1.19 (0.70 to 2.01)	0.52
3	8 (5)	8 (5)	1.03 (0.39 to 2.71)	0.95
4	3 (2)	5 (6)	1.72 (0.49 to 6.08)	0.40
Procedural complications^a^	8 (6)	19 (13)	2.51 (1.09 to 5.76)	0.03
Major complications	6 (4)	14 (9)	–	
Hypotension	3 (2)	9 (6)	–	
Hypoxia	3 (2)	7 (5)	–	
Aspiration	1 (1)	3 (2)	–	
Arrhythmia	2 (1)	0 (0)	–	
Dental trauma	0 (0)	1 (1)	–	
Esophageal intubation	0 (0)	3 (2)	–	

Raw data presented as number (%). DL, direct laryngoscopy. ^a^Complications with frequencies of 0 are omitted from the table.

When we compared the first intubation in repeatedly intubated patients with age-matched and sex-matched singly intubated patients, we found no baseline differences except for an increased incidence of missing mouth opening in the repeatedly intubated group (Table 2). However, use of adjuncts to DL was significantly more common in the first intubation of repeatedly intubated patients (11% vs. 1%, *P* <0.001) and repeatedly intubated patients were significantly more likely to require ≥3 intubation attempts (11% vs. 6%, *P* = 0.03). There were no other differences between groups, except for an increased incidence of missing Cormack–Lehane grade in the repeatedly intubated patients.

**Table 2:** **Comparison of baseline characteristics and outcomes between the first intubation in repeatedly intubated (exposed) patients with matched, singly intubated (unexposed) patients**

**Characteristic**	**Repeatedly intubated**	**Singly intubated**	***P*** **value**
	**(** ***n*** **= 151)**	**(** ***n*** **= 302)**	
**Baseline airway assessment**			
Mallampati class			0.39
I or II	85 (56)	162 (54)	
III or IV	20 (13)	55 (18)	
Missing	46 (30)	85 (28)	
Mouth opening			0.02
< 2 cm	13 (9)	9 (3)	
≥ 2 cm	110 (73)	245 (81)	
Missing	28 (19)	48 (16)	
Thyromental distance			0.09
< 2 fingers	61 (40)	98 (32)	
≥ 2 fingers	53 (35)	137 (45)	
Missing	37 (25)	67 (22)	
Neck range of motion			0.33
Normal	107 (71)	213 (71)	
Limited	6 (4)	15(5)	
Cervical spine collar	7 (5)	26 (9)	
Missing	31 (21)	48 (16)	
Provider anticipated difficulty	37 (27)	75 (25)	0.70
**Difficult intubation**			
Intubation attempts			
1 or 2	134 (89)	285 (94)	0.03
≥ 3	17 (11)	17 (6)	
Rank of final intubator			0.77
Fellow	98 (70)	210 (70)	
Resident or other	28 (21)	64 (21)	
Attending	16 (9)	28 (9)	
Adjunct to DL used^a^	16 (11)	3 (1)	<0.001
Cormack–Lehane grade			0.02
1	91 (60)	201 (67)	
2	33 (22)	74 (25)	
3	3 (2)	16 (5)	
4	3 (2)	4 (1)	
Missing	16 (11)	7 (2)	
**Procedural complications** ^**b**^	8 (6)	33 (12)	0.07
Major complications	6 (4)	28 (9)	0.06
Hypotension	3 (2)	17 (6)	0.09
Hypoxia	3 (2)	13 (4)	0.28
Arrhythmia	2 (1)	3 (1)	1.00
Aspiration	1 (1)	4 (1)	0.69

Data presented as number (%). *P* values are derived from generalized estimating equations (dichotomous outcomes) or overall comparisons from multinomial logistic regression (categorical outcomes). DL, direct laryngoscopy. ^a^Adjuncts to DL included use of a gum elastic bougie, fiberoptic bronchoscopy, an intubating laryngeal mask airway, an Airtraq® (Prodol Ltd., Vizcaya, Spain) laryngoscope and a King® (Kingsystems, Noblesville, IN, USA) supraglottic airway. ^b^Complications with frequencies of 0 are omitted from the table.

## Discussion

In this group of repeatedly intubated patients, procedural complications were more frequent during last intubations compared with first intubations. Interestingly, this occurred despite no difference in any measurable marker of technical difficulty. Because our work is observational, we cannot comment on causality. However, an increase in complications without a corresponding change in technical difficulty suggests that patient physiologic factors such as deconditioning or sequelae of critical illness may drive this effect, rather than anatomic airway factors. Consistent with this hypothesis, the risk of complications increased with longer times from extubation to reintubation. Regardless of mechanism, when preparing for emergent, out-of-operating room intubation in a previously intubated patient, providers should prepare for an increased risk of procedure-associated hypotension and hypoxia. These complications are clinically important. Peri-intubation hypotension [23-26] and hypoxia [27,28] have been associated with increased mortality, even after controlling for other measures of disease severity. Both of these complications are also potentially preventable through appropriate use of intravenous fluid for preload optimization, vasopressors and preoxygenation [29-31].

We contrast our findings with those reported by Menon and colleagues, who found in a retrospective study that technical difficulty and complication rates did not differ between initial and subsequent intubations [2]. While our findings are consistent with theirs with regard to difficulty of reintubation, we found an increased rate of procedural complications for last intubations. Several differences in study design may explain this difference. First, our data collection was prospective and self-reported by providers shortly after intubation. Therefore, we were able to included information that would not be otherwise available in the medical record. By contrast, Menon and colleagues conducted a retrospective chart review. This may be less susceptible to reporting bias, but may also lack sufficient detail to adequately capture important complications. Second, intubations in our study were performed in the in-hospital setting by physicians, whereas prehospital providers performed almost one-half of the initial intubations in Menon and colleagues’ work.

The complication rate we observed is consistent within the range of 5 to 24% that has been described by other authors for emergency intubations in general [9-16], but is lower than those described by Mort, who reported in a smaller study that 72% of patients suffered complications on reintubation following unplanned extubations, and virtually all patients were reintubated within 6 hours [32]. This is notably different from our patient population, where only 8% were reintubated after unplanned extubation. Previous intubation may make subsequent reintubation more difficult because of anatomic changes such as laryngeal edema or airway trauma, which may not be predicted or assessed accurately with the traditional measures of predicted difficulty that we included in our study. However, laryngoscopic evaluation has shown that it takes several weeks for laryngeal trauma caused by endotracheal tube placement to resolve, and even 4 weeks after extubation one-half of the patients exhibit laryngeal edema [8]. Therefore, in so far as anatomic factors contribute to difficulty, it may be unsurprising that we did not find any change in difficulty between early and late reintubation.

Alternatively, patients may have been physiologically sicker or deconditioned later in their hospitalizations and therefore more susceptible to procedural complications. Previous authors have described comorbid conditions such as kidney disease and respiratory failure as risk factors for procedural complications during intubation [21,23]. Indeed, when we adjusted for the time from antecedent extubation to reintubation, we observed an increased risk of procedural complications in the patients who underwent late reintubation (≥72 hours after extubation). This is suggestive that physiologic changes are the major driver of the increased complications, rather than anatomic changes such as laryngeal trauma or edema, which one would expect to decrease over time. Our registry does not include sufficient data to calculate and control for measures of overall physiologic status or disease severity such as a Simplified Acute Physiology Score or an Acute Physiology and Chronic Health Evaluation score. Importantly, the time between antecedent extubation and reintubation in our study ranged from hours to weeks, sometimes even months. This raises the possibility that some of the reintubations included in our analysis actually occurred during a different admission or disease presentation than the initial intubation. Indeed, eight reintubations occurred in the ED, supporting this assertion.

Interestingly, we found that adjuncts to DL were used more frequently in reintubated patients, and these patients were more likely to undergo ≥3 intubation attempts. It is possible that patients who ultimately go on to require reintubations are initially more difficult to intubate. This could be related to anatomic or patient characteristics that predispose these patients to be at high risk of extubation failure. Alternatively, use of airway adjuncts such as laryngeal mask airways and repeated intubation attempts may be injurious in some patients, for example by increasing the incidence of laryngeal trauma, subsequent laryngeal edema and extubation failure. To our knowledge, no other studies have linked initial intubation conditions to the risk for subsequent failed extubation. Given the observational nature of our study, we view these observations as hypothesis generating and exploratory, but refrain from drawing firm conclusions. Although it is theoretically possible that use of adjuncts to DL might be injurious, these adjunct devices can be life saving in emergency airway management.

Our work has important limitations. First, procedural complications and details of the intubation procedure were self-reported and may be subject to recall or reporting bias on the part of the provider. However, we do not anticipate a differential in either of these biases between patients’ first and last intubations, and so we do not believe this would have led us to make a type I error. Further, by using self-reported data we were able to assess clinically important details that might not otherwise be available in a medical record review. When data entry forms were crosschecked against patient medical records, we noted few missing clinically relevant data with many additional procedural complications that were self-reported but not recorded in the medical record. Another limitation is that, since our work was retrospective, we could not perform an *a priori* power calculation and *post hoc* power calculations have little statistical meaning or utility [33]. We therefore cannot accurately estimate the risk of having made type II errors. Additionally, in our comparison of reintubated patients with matched unexposed patients, a portion of our unexposed population may have undergone subsequent intubations that occurred after our data collection ended. This would decrease our observed effect size and would increase our risk of a type II error. Finally, this study was performed in a single large, urban academic medical center with critical care fellows performing most intubations, which may limit the generalizability of our findings to other settings and patient populations.

## Conclusions

We find that emergent reintubation is associated with an increased risk of clinically important procedural complications when compared with first intubation. When approaching these patients, providers should anticipate the risk of periprocedural hypotension and hypoxia, and prepare for these complications.

## Key messages

- Procedural complications were more frequent during last intubations compared with first intubations.
- This occurred despite no difference in any measurable marker of technical difficulty.
- When approaching these patients, providers should anticipate the risk of periprocedural hypotension and hypoxia, and prepare for these complications.

## Abbreviations

DL: Direct laryngoscopy
ED: Emergency department
